# Accumulation of Vesicle-Associated Human Tau in Distal Dendrites Drives Degeneration and Tau Secretion in an *In Situ* Cellular Tauopathy Model

**DOI:** 10.1155/2012/172837

**Published:** 2012-01-17

**Authors:** Sangmook Lee, WonHee Kim, Zhihan Li, Garth F. Hall

**Affiliations:** Department of Biological Sciences, Center for Cellular Neuroscience and Neurodegeneration Research, University of Massachusetts Lowell, Lowell, MA 01854, USA

## Abstract

We used a nontransgenic cellular tauopathy model in which individual giant neurons in the lamprey CNS (ABCs) overexpress human tau isoforms cell autonomously to characterize the still poorly understood consequences of disease-associated tau processing *in situ*. In this model, tau colocalizes with endogenous microtubules and is nontoxic when expressed at low levels, but is misprocessed by a toxicity-associated alternative pathway when expressed above levels that saturate dendritic microtubules, causing abnormally phosphorylated, vesicle-associated tau to accumulate in ABC distal dendrites. This causes localized microtubule loss and eventually dendritic degeneration, which is preceded by tau secretion to the extracellular space. This sequence is reiterated at successively more proximal dendritic locations over time, suggesting that tau-induced dendritic degeneration is driven by distal dendritic accumulation of hyperphosphorylated, vesicle-associated tau perpetuated by localized microtubule loss. The implications for the diagnosis and treatment of human disease are discussed.

## 1. Introduction

Alterations of the microtubule-associated protein (MAP) tau that affect tau:tau and tau:tubulin interactions are a key factor in the cytopathogenesis of neurodegenerative syndromes involving tau (tauopathies) [1–3] reviewed in [4, 5]. Phosphorylation at proline-directed sites flanking the microtubule binding repeat (MTBR) domain is inhibitory to MT binding, and ultimately causes the formation of tau aggregates [6] and progressive loss of MTs and neurodegeneration in tauopathy models [7–12]. The extent to which this occurs is likely a key determinant of whether tau is playing a normal or pathological role [4, 13]. Exonic tau mutations responsible for familial tauopathies cause hyperphosphorylation, a reduction in MT binding, and an increased tendency to aggregate [14–17], suggesting that a causal link exists between these features and toxicity. Although mutant tau isoforms are more toxic than wild-type (WT) isoforms when expressed *in situ *[7], the patterns of degeneration produced by WT and mutant tau species are very similar, even in disease models using identified neurons [18], suggesting that the cytoplasmic accumulation of hyperphosphorylated, non-MT-bound tau is a common feature that drives toxicity.

Since the tau MTBR forms the core of the neurofibrillary aggregates characteristic of tauopathies and is the site of most exonic mutations that drive familial tauopathies [6, 19, 20], the modulation of tau:MT and tau:tau interactions via tau phosphorylation [1, 3, 13, 21] or cleavage [22–25] to form toxic oligomers or aggregates [26] has become the central research focus in studies of tau-induced toxicity and tauopathy cytopathogenesis. However, very little is known about how tau aggregates are produced and distributed in the somata and dendrites of neurons in situ, making it difficult to establish the sequence and causality of cellular events that lead to neurodegeneration. The role of aggregation is further obscured by findings that cleavage fragments of the amino terminal “projection” domain of tau [27–30] can also mediate tau toxicity via a separate pathway [30, 31]. This pathway involves caspase [32] and calpain [33] activation and can mediate toxicity caused by extracellular agents, such as beta amyloid [34]**·** We now know that tau misprocessing also involves the interaction of tau with lipid raft-associated proteins at the plasma membrane via the tau N-terminal domain [35, 36], which becomes phosphorylated at tyrosine 18 (Y18) [37]. Abnormal tau-actin interactions [38] and the misrouting of tau to the Golgi [39] in tauopathy suggest that an abnormal association with the plasma membrane and/or “trafficking” vesicles derived from it may also play a role in tau-mediated toxicity. Recent findings that tau is secreted to [40, 41] and taken up from [42] the extracellular space and that it can be transported transneuronally *in situ* [40, 43] make it necessary to distinguish cell autonomous from interneuronal effects of tau misprocessing. In this study, we used the unique advantages of the lamprey ABC tauopathy model, in which tau is expressed cell autonomously in identified neurons (ABCs), to characterize tau misprocessing at the cellular level in neurodegeneration in situ.

We use confocal microscopy and the high spatiotemporal resolution offered by the ABC tauopathy model to make a detailed correlation between the expression level, intracellular distribution, phosphorylation state, and secretion of human tau isoforms relative to the distribution and stability of MTs. We previously used this model to show that tau phosphorylated at Y18 (9G3+ tau) becomes associated with vesicle-like particles and accumulates in ABC dendritic tips [18, 41], where it causes distension and ultimately degeneration [8, 9, 18, 41]. In this study, we address the role(s) played by (a) tau-MT binding (and the phosphorylation of sites known to affect tau:MT binding) and (b) the effects of tau expression level and somatodendritic distribution in producing the toxic effects caused by tau expression on the neuronal cytoskeleton *in situ. *We show that exogenous tau expression above the level needed to saturate dendritic MTs results in the redistribution of specific phosphotau species (9G3/12E8 tau) distally to dendritic tips, dendritic MT loss in the vicinity of tau accumulation, and tau secretion from tau accumulation sites with ongoing or recent MT loss. In addition, we characterized the morphology of exocytosing vesicles involved in tau secretion and identified proteins that colocalize with tau as it is secreted. We propose that non-MT-associated tau accumulation activates an alternative tau processing pathway involving abnormal endosome formation, centrifugal dendritic transport, and secretion of vesicle-associated tau and suggest that this may play a key role in the cellular and intercellular pathogenesis of human tauopathies.

## 2. Methods

### 2.1. Tau Expression

We used the pEn123c and pEn1234c plasmids encoding green fluorescent protein-(GFP-) tagged wild-type human tau 3R0N and 4R0N isoforms (resp.) for most of these experiments. We also used P301L tauopathy mutant of 4R0N and a double pseudophosphorylation mutant at S262D and S356D in the MTBR of 3R0N that were derived from the corresponding parent WT plasmids [18, 41]. Plasmids encoding lamprey neurofilament (NF180) [44] or GFP [45] served as expression controls. All plasmids used the CMV promoter to drive exogenous protein expression. Microinjection of plasmids was performed as described [8], using 1 mg/mL of plasmid mixed in microtubule stabilizing buffer.

### 2.2. Immunochemistry

**Figure 1:**
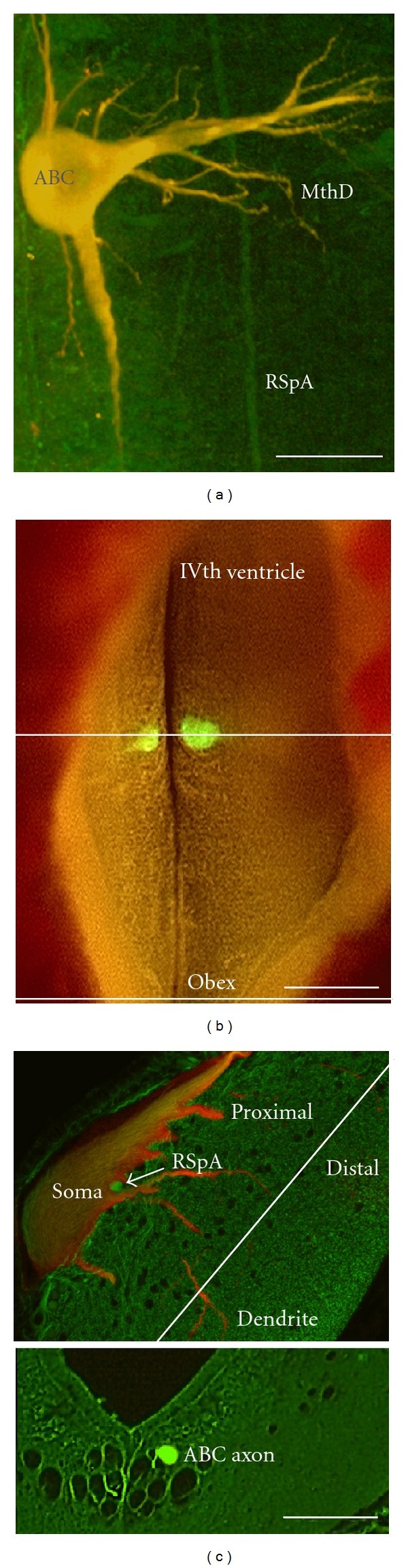
ABC morphology. (a) shows a confocal micrograph of a wholemounted lamprey brain with an ABC expressing low levels of human tau and showing its characteristic gross morphology. Note that tau (red channel) is evenly codistributed with tubulin (DM1A-green channel), resulting in a yellow appearance. Tubulin immunolabel in axons from large reticulospinal neurons other than ABCs (RSpA) can be seen in whole mount (a) and in cross-section (c), and a Mauthner's cell lateral dendrite can be seen in the whole-mounted preparation (MthD). (b) Dorsal view of a live lamprey brain with a pair of ABCs expressing exogenous GFP-tagged protein. Ten-micron-thick transverse paraffin sections were taken through the soma and dendritic fields of tau-expressing ABCs (upper line) and though ABC axons in the caudal hindbrain near the obex (b, bottom line). (c) Somatodendritic section through an ABC expressing low levels of Tau24 (4R0N tau). Both the whole- mount in (a) and the top section shown in (c) were immunostained with the mAb DM1A for total alpha-tubulin and anti-GFP for total exogenous tau. The bottom image is of a transverse section though ABC axons in the caudal hindbrain near the obex (at the level indicated in (b), bottom line). Scale bars: (a) 100 *μ*m, (b) 200 *μ*m (c) 50 *μ*m.

Immunohistochemical analysis of transverse sections through ABC somata, dendrites, and axons (Figure 1) was performed using standard enzyme-linked and immunofluorescence protocols [8, 18, 41, 47, 48]. After 10 days following plasmid injection, brains were reexposed under fluorescence to identify ABCs expressing GFP-tagged exogenous proteins and then fixed with FAA (10% formalin, 10% glacial acetic acid, and 80% ethanol), dehydrated, embedded in paraffin, and sectioned at 10 *μ*m. Rabbit polyclonal anti-GFP (1 : 200, Invitrogen) were used to detect the expression of GFP-tagged tau or NF-180. HRP-linked staining with diaminobenzidine (Sigma) was used on every 7th section to reveal immunopositive ABCs, with the remaining sections used for immunofluorescence. Sections were deparaffinized, rehydrated, quenched with H_2_O_2_, boiled for 10 min in pH 9 and 10 mM Tris-EDTA buffer to unmask antigen, and incubated in primary antibody overnight at 4°C. For immunofluorescence staining, sections were quenched 10 minutes in 1% sodium borohydride/TBS.

### 2.3. Antibodies

We used the following primary antibodies: PHF1 (1 : 200), Tau1 (1 : 400), Tau5 (1 : 400), Tau7 (1 : 400), Tau12 (1 : 400), Tau46 (1 : 100, Sigma), 9G3 (1 : 30), AT180 (1 : 200, Pierce), DM1A (1 : 500, Sigma), TUB1 (1 : 500, Sigma), and 611B (1 : 500, Sigma). Tau1, Tau5, Tau7, and Tau12 antibodies were kindly provided by Dr. Lester Binder (Department of Cell and Molecular Biology, Northwestern University Medical School, Chicago, IL, USA). PHF1 was a generous gift from Dr. Peter Davies (Department of Pathology, Albert Einstein of College of Medicine, Bronx, NY, USA). 9G3 was provided by Dr. Gloria Lee (University of Iowa, Iowa City, IA). Polyclonal Abs against human fyn (1 : 50, Santa Cruz), LC3 (1 : 30, Abgent), and COX1 (1 : 200, MitoScience) were used to label the highly conserved eponymous lamprey homologues of fyn (92% identity), LC3 (73% identity), and COX1 (64% identity).

### 2.4. Data Analysis

**Figure 2:**
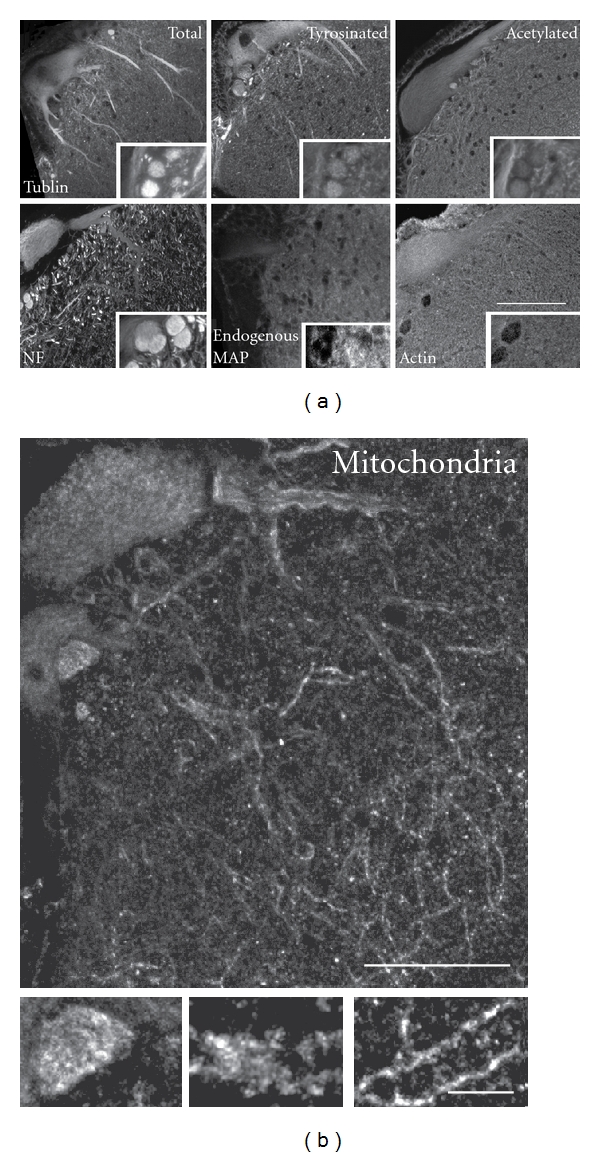
Somatodendritic cytoskeletal elements in ABCs. (a) Somatodendritic and axonal distribution of cytoskeletal elements in ABCs are shown in transverse sections through ABC somata and dendrites immunostained for total, dynamic, and stably polymerized tubulin with axonal profiles shown in insets (top). The distributions of the major neurofilament protein NF180, a tau-like endogenous MAP, and a mitochondrial marker (COX1) are similarly displayed (bottom). MTs are the dominant dendritic element (especially tyrosinated tubulin-rich MTs in large proximal dendrites), whereas acetylated MTs and NFs predominate in the axon. An endogenous, axonally localized tau-like protein occurs in sea lampreys, but is poorly expressed in ABCs. (b) Mitochondria are typically distributed evenly throughout ABC dendrites and are somewhat more concentrated in axonal profiles (inset). Scale bars: (a) 100 *μ*m, (b) 50 *μ*m, (b) inset 10 *μ*m.

Immunofluorescence intensity was measured in multiply immunolabeled sections containing somatodendritic profiles of tau-expressing ABCs. Confocal images were analyzed using either ImageJ or Volocity (ImproVision) to generate image stacks. In some cases, densitometric analysis of nonconfocal images of DAB-revealed adjacent sections from the same brain was performed. For quantitative assessment of exogenous tau immunolabel intensity or for comparisons between different phosphotau epitopes, the immunolabel density at the point of dendritic degeneration (POD) was normalized to 100%, with localization along dendrites defined in terms of distances from the POD (distal = plus). For the calculation of tau:tubulin intensity ratios and for comparing “absolute” levels of tau expression (e.g., to assign ABCs to “low” and “high” tau expression categories), we compared tau (GFP tag) versus tubulin (DM1A) immunolabel intensities in confocal stacks of multiply immunostained sections, which were scored relative to DM1A neuropil immunolabel in that location of the section using the same PMT settings. Intensity of tubulin (DMIA, TUB1, 611b) immunolabel was scored relative to the neuropil surrounding ABC axonal, dendritic, and somatic profiles. The cellular elements of “neuropil” consist mainly of small axon cross/longitudinal profiles that stain relatively uniformly for the probes shown in Figure 2 and are unaffected by exogenous protein expression in ABCs. All comparisons of immunolabel intensity were done using a two-sample *t*-test or one-way ANOVA as appropriate; such data are expressed as mean ± SEM, and significance was expressed at *P* values as noted. Quantitative analyses that required the categorization of data sets according to defined criteria, such as “high” versus “low” tau expression (as defined in the Results section), were analyzed using the chi-square test. Ratios of phosphotau to total tau was calculated using the “sync windows” and “sync measure” 3D plug ins of ImageJ. Quantitative comparisons of axonal versus dendritic tau immunolabel intensity were made using the “Measure RGB” plugin. This was only done in “low” tau expressing ABCs and was restricted to sections where both axonal and dendritic profiles were unambiguously present. Unbiased comparison of tau label intensity between axon profiles (which invariably extended through the entire section) with dendritic profiles which occupied varying proportions of the *Z* axis was accomplished by normalizing tau to tubulin levels in each sample. Quantitation of DAB product for tubulin versus tau immunolabel ratios in the axons of tau-expressing ABCs and adjacent axonal profiles of similar size was performed on 10 sets of 4 adjacent sections in the obex. Ratios between tau versus tubulin immunostaining intensity were performed by comparing the intensity of label between the red and green color channels on a per voxel basis using the ratio channel function of Volocity 4.3.

### 2.5. Colocalization Analyses

Semiquantitative comparisons of the degree of colocalization to MTs between (a) dendritic GFP-tau label in low- and high-expressing ABCs (Figure 4) and (b) markers of high- (tau1, tau5) and low- (9G3) MT-affinity tau species (Figures 6 and 7) was performed on stacks using ImageJ “colocalization highlighter” at a common threshold value. Images in which only the colocalized voxels are shown (such as was done with the phalloidin label in some of the images in Figure 7) were obtained by substituting the “colocalized points” 8-bit file generated by colocalization highlighter for the original channel in the final image, which was produced using the ImageJ plugin “3D Viewer”.

## 3. Results

### 3.1. ABC Cytoskeleton

The composition of the somatodendritic and axonal cytoskeleton in lamprey ABCs is typical of large projection neurons in vertebrate central nervous systems [8, 47, 48]. Somatodendritic MTs consist of both stably polymerized and dynamic tubulin in ABCs, especially in large proximal dendrites (5+ microns diameter). Total and tyrosinated (i.e., recently polymerized) alpha-tubulin levels are highest in such dendrites and both are clearly above neuropil levels in all but the smallest dendrites. Levels of acetylated (stably polymerized) tubulin are comparatively lower, with the largest dendrites staining near neuropil levels and the smaller ABC dendrites staining below. Tubulin immunolabel intensity in ABC axons is generally similar to those seen in the soma and typically were not as strong as those of dendritic tubulin. NF immunolabel intensity (LCM3 [44]) is above neuropil levels in large proximal dendrites, declining distally to below neuropil in the smallest dendrites (Figure 2). NF staining was stronger in axons than in somata and dendrites [43, 44], and axonal NF label was stronger than that of tubulin. This is to be expected given that NF to MT frequency in large axons such as those of ABCs is as high as 20 : 1, and up to 3x higher than that of small axon profiles [46, 49, 50]. Cross reaction of mAbs T46 and Tau7 (not shown) to the relatively well-conserved tau C terminus [51] identified an axonally targeted endogenous lamprey MAP (lamprey tau). Lamprey tau is concentrated in small axons, is relatively low in ABC axons, and is virtually absent from ABC somata and dendrites (Figure 2) and so was not considered to be a significant factor in the distribution and MT-binding of exogenous tau species (see below).

### 3.2. Expression of Exogenous Proteins in ABCs

**Figure 3:**
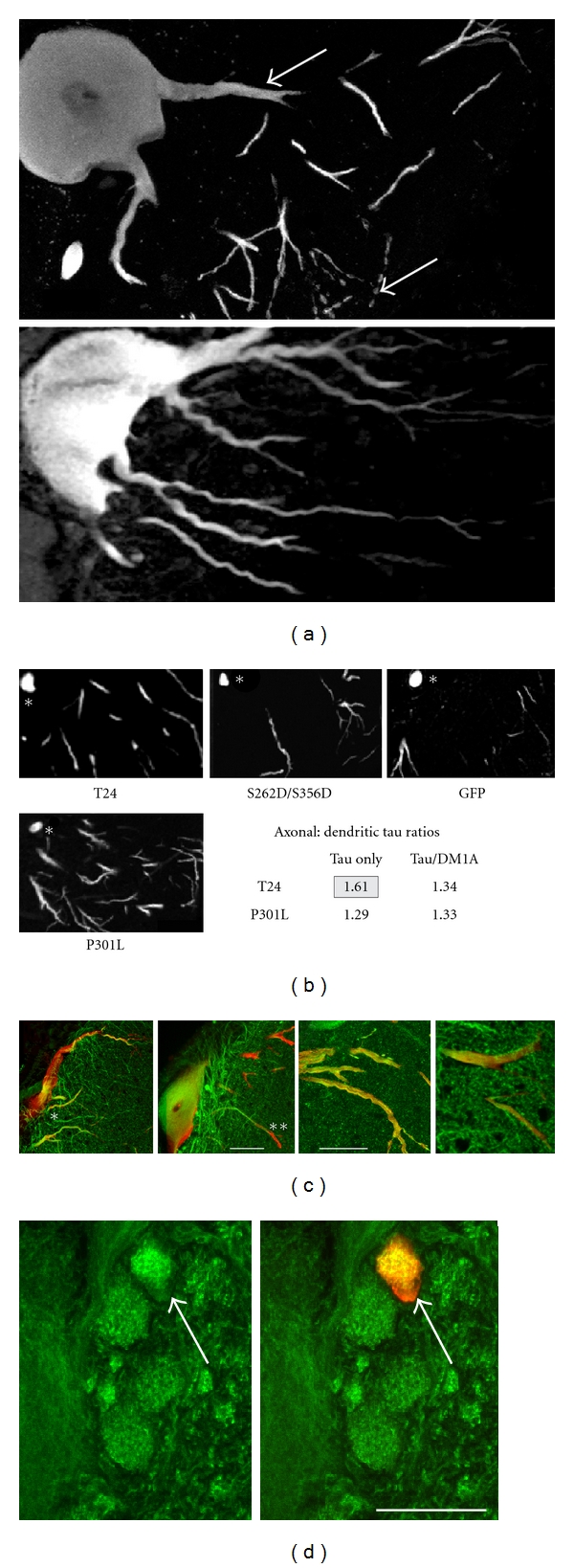
The intracellular distribution of exogenous human tau versus controls. (a) Somatodendritic sections through ABCs expressing tau (top) and an expression control GFP (bottom) showing the selective axonal (asterisk) and distal dendritic distribution (arrows) of tau versus the relatively even distribution of exogenous protein. (b) Tau immunolabel is somewhat more concentrated in the proximal axon than in proximal dendrites in low expressing ABCs. However, any selective localization to the axon (asterisk) is slight and appears to be lost entirely when normalized to MT immunolabel. (c) Examples of double-labeled sections through the somata and dendrites of ABCs exhibiting low (left) and high (left center) tau expression illustrate the characteristic changes in tau distribution with expression level and their relationship with dendritic beading and MT distribution relative to NF-180 expression controls. Tau (red) and tubulin (green) are colocalized in the proximal dendrites of low expressing cells (asterisk), but tau immunolabel becomes significantly more distally located than that of tubulin in ABCs expressing higher tau levels (right, double asterisks). Note that the expression controls do not affect MT distribution when NF-180 is expressed in ABCs (right panels). (d) Human tau expression appear to “bundle” endogenous MTs (compare green channel label at arrows), even when tau expression is above MT saturation, unlike the situation with dendrites. Scale bars: 50 *μ*m.

Previous comparisons of the effects of expressing human tau via plasmid microinjection [8, 9, 45, 52] versus a control proteins (lamprey neurofilament NF180) showed that toxicity due to overexpression is tau specific. Here we used NF180 and GFP to control for nonspecific overexpression effects on MT integrity and other cytoskeletal changes, especially in the proximal dendrites and axon. Expression of GFP alone resulted in a relatively even distribution of anti-GFP immunolabel, while the distribution of exogenous NF180 resembled that of endogenous NF180 (Figure 3).

### 3.3. Distribution of Exogenous Tau between the Axon and Dendrites Is Modulated by Tau Expression Levels in ABCs

**Figure 4:**
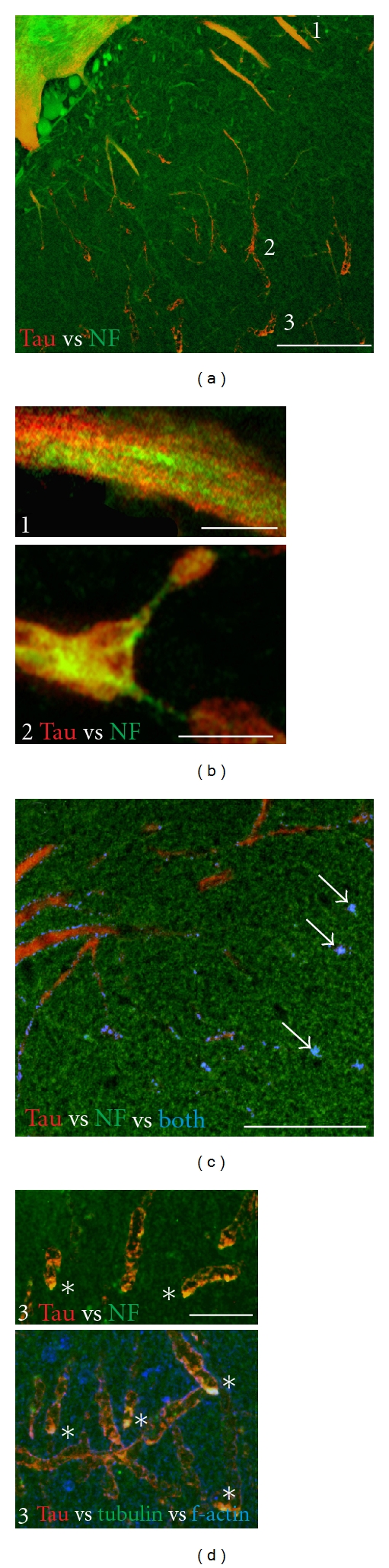
Exogenous tau expression can redistribute and sequester endogenous cytoskeletal elements. (a) Expression of exogenous human tau in ABCs at moderate expression levels can result in the colocalization of tau with endogenous NFs where tau accumulates in dendritic tips. Note that NF immunolabel in nonexpressing ABCs decreases to neuropil levels in the distal dendrites and that aggregates of NF immunolabel occur only with tau co-localization. The locations of large proximal (1) and smaller distal dendrites (2, 3) are indicated. (b) (1) top: NF distribution in a large dendrite of an ABC expressing tau at moderate levels is independent of tau (red channel). (2) bottom: significant changes in NF distribution in more distal dendrites (location 2 in (a)), with extensive overlap between the red (tau) and green (NF) channels and relocalization of NFs into punctate deposits (carets) that accumulate in dendritic beads (arrow). (c) shows tau (red) and fluorescently tagged phalloidin (filamentous actin, green) distribution. Colocalized voxels can be found in distal dendrites (blue channel). (d) shows high magnification views of co-localized tau (red channel) with NF protein (top), with tubulin (bottom, green channel), and f-actin (bottom, blue channel) in amorphous aggregates (asterisks) at the distal tips of the ABC shown in (3). Note that the white aggregates shown in the bottom image (asterisks) are the result of colocalization of tau with both tubulin (DM1A) and f-actin (phalloidin). Scale bars: (a) 50 *μ*m, (b) 10 *μ*m, (c) 20 *μ*m, (d) 5 *μ*m.

**Figure 5:**
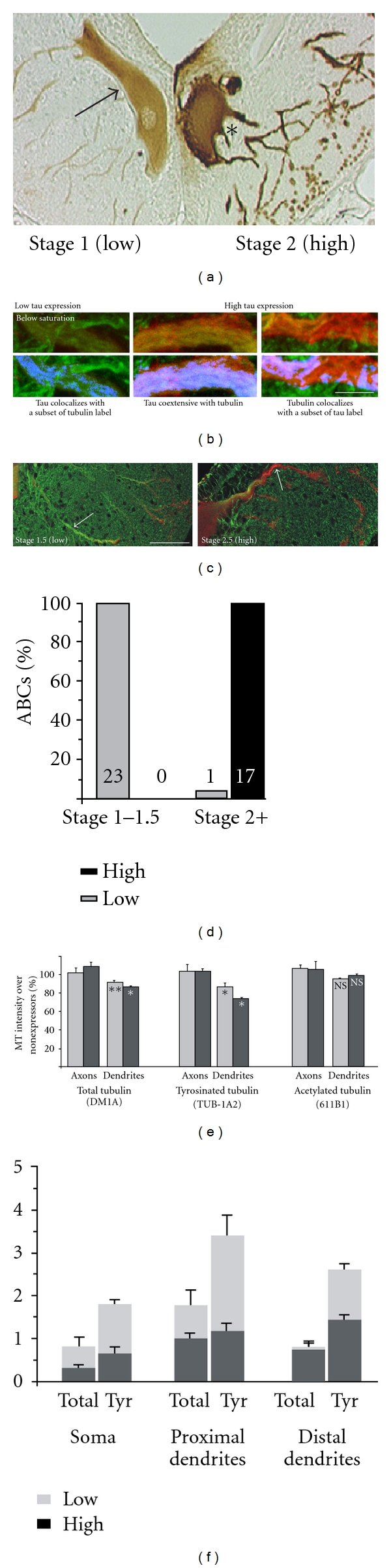
The state of tau:tubulin colocalization defines the threshold for tau-induced toxicity in ABCs. (a–c) illustrate the correlation between tau:tubulin colocalization pattern and neurodegenerative stage [46]. (a) shows a typical example of low (left cell) and high (right cell) expression in the same section, permitting a direct comparison of staining intensity (DAB). Note that much of the heaviest tau staining in the high expressing cell occurs near the plasma membrane instead of colocalizing with the dendritic cytoskeleton. In particular, the tau label in the low expressing cell excludes the ventral soma, while this region stains heavily in the high expressing cell. (b) Images of tubulin (green) and tau (red) immunolabel from proximal dendrites illustrate the criteria used to assign ABCs to “low” and “high” expressing categories as described in the Methods section. Bottom images show colocalized voxels (colocalization highlighter, ImageJ) in the blue channel. With low tau expression (left and center pairs), exogenous tau was largely colocalized to endogenous MTs in the proximal dendrites, whereas in “high” tau expression, much of the tau label was distributed in areas devoid of tubulin label. (c) A comparison between the distribution of tau (GFP tag, red channel) and tubulin (DM1A, green channel) immunolabel in somatodendritic profiles of low-stage (left) and high-stage (right) ABCs. This clearly shows the relationship between MT saturation state with tau in large proximal dendrites and the stage of tau-induced degeneration. Note that only the distalmost dendrites of the ABC at the left (swollen, but not beaded) have accumulated high levels of tau, whereas the cell at the right is exhibiting both dendritic beading and some tau secretion (inset, asterisk). (d) The correlation between the level of tau expression and tau-induced degeneration was found to define the threshold level of tau expression above which tau is toxic to ABCs. The correlation between ABCs that exhibited “above saturation” levels of tau in proximal dendrites and those exhibiting significant beading and fragmentation of tertiary and quaternary dendrites (Stage 2 or greater of degeneration) was virtually without exception and was highly significant (*P* ≪ 0.001, chi-square test). (e) Quantitative analysis of the effect of tau expression on total, tyrosinated and acetylated tubulin is shown for the axon (left) and dendrites (right). Tau overexpression had little effect on axonal tubulin levels but significantly reduced both total and tyrosinated tubulin levels and most prominently in normally MT-rich proximal dendrites (right). No effect was seen for acetylated tubulin staining in the dendrites (not shown). Scale bars: (b) 10 *μ*m, (c) 50 *μ*m.

We used the distribution of total exogenous human tau (GFP tag) relative to that of endogenous tubulin (DM1A) immunolabel in proximal dendrites to sort ABCs into “low-expressing” and “high-expressing” groups (Figures 3 and 5). In “low-tau” ABCs, the axon and proximal dendrites exhibited the strongest staining in a pattern that was similar to that of tubulin (Figure 3). This distribution is consistent with the known function of tau as a MAP and is the expected pattern if tau is primarily binding directly to MTs. There was a slight preferential distribution of tau to the proximal axon relative to proximal dendrites (Figure 3(b)), but this was only seen at low expression levels in comparisons between raw GFP-tau levels in WT T24-expressing ABCs versus GFP alone. The difference between WT and P301L tau distribution disappeared when axon:dendrite tau ratios were normalized to tubulin levels to account for differences in *z*-axis profile dimensions between axons and dendrites. Preferential axonal staining did not appear to involve the ability to bind MTs directly, since it was still present with the S262/356D mutant [50]. By contrast, with “high” tau expression, tau tended to accumulate in perimembranous regions of the soma and in distal dendrites (Figures 3(c) and 4). Moreover, tau in high-expressing ABCs was preferentially localized to dendritic tips and was thus much more distally located than dendritic tubulin staining. The most striking difference between the “low” and “high” distributions of tau was that in the latter case the areas of highest tau accumulation were devoid of MTs, whereas tau and tubulin were colocalized with “low” tau expression (Figure 3(c)).

### 3.4. High Tau Expression Causes Redistribution of Somatodendritic NFs and f-Actin in ABCs

In low tau-expressing ABCs, exogenous tau was distinct from that of endogenous NF-180 and filamentous actin (f-actin) in all parts of the soma and dendrites, with tau being colocalized with MTs (Figures 4(a) and 4(b)). High levels of tau expression did not affect this pattern in the proximal dendrites of high-expressing ABCs; we found that colocalization of NF protein and f-actin with tau did occur in the distal dendrites of some high tau-expressing cells. This was most noticeable at the sites with the largest accumulations of non-MT-associated tau, which were typically in the vicinity of the POD or the dendritic tips, depending on the extent of dendritic degeneration. In the case of endogenous NFs, tau-associated NF180 protein was displaced distad of its typical location; this is readily apparent as there are relatively few NFs in the dendrites of non-tau expressing lamprey neurons [46, 48].

### 3.5. Tau Distribution and Toxicity in ABCs Is Modulated by the Saturation of Tau Binding Sites on Endogenous MTs

Low tau expression is defined as being below or at the level of tau:MT “saturation” (tubulin is 100% colocalized with tau, and tau is at least 80% colocalized with tubulin) with all tau being colocalized with tubulin in ABC proximal dendrites (Figure 5). High expression is defined as tau expression above the level needed to saturate MTs in ABC proximal dendrites. Low-expressing ABCs nearly always (Figure 5(c)) show low-stage (1 or 1.5) tau-induced degeneration. This definition is consistent with the staging scheme of tau-induced degeneration described in an earlier study [52] in which stage 1 was defined by the filling of dendritic profiles with even tau label, and higher levels of tau expression (stages 2-3) were characterized by the distal to proximal extent of dendritic degeneration. ABCs staged as “high expressing” (i.e., stage 2 and above) by this method were almost perfectly cosegregated with those scored as having proximal dendrites “above saturation” (Figure 5(c)). 

### 3.6. Tau Redistribution with High Expression Is Correlated with Microtubule Loss and Dendritic Degeneration

**Figure 6:**
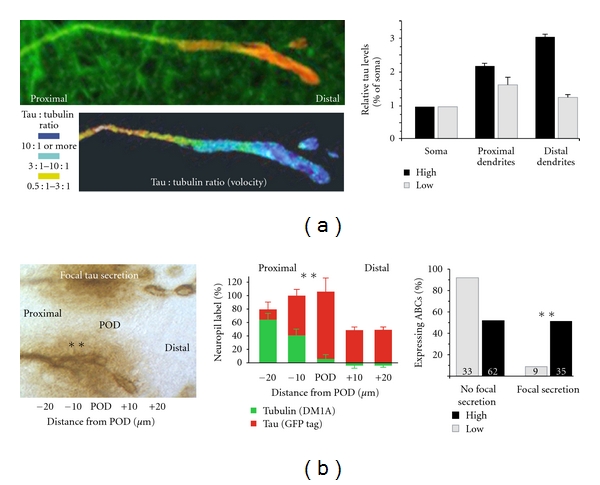
Accumulation of exogenous tau in the distal dendrites of high tau expressing ABCs is correlated with localized MT loss in distal dendrites and tau secretion. (a) left: Dendrite showing distal tau accumulation relative to tubulin level as an anti-GFP: DM1A signal ratio (Volocity), right: a graph summarizing the selective distal dendritic tau accumulation as a function of tau expression level (Bars: SEM). (b) left: A high-expressing ABC shows extensive tau secretion (asterisks) from morphologically intact dendrites near and immediately proximal to the POD. The graph to the right shows the relative intensity of tubulin (green, DM1A) versus tau (red, GFP) as a function of distance from the POD. Intact dendrites immediately proximal to the POD show a combination of recent MT loss and tau accumulation. Tubulin immunolabel levels were normalized to those in adjacent neuropil as described in the Methods section. Right: Low and high expressing ABCs were scored (+/) for degeneration and the presence of peridendritic secreted tau deposits. High tau expression (Stage 2+) was strongly correlated with secretion (bottom, asterisks *P* < 0.01, chi-square test).

The most dramatic effect of high tau expression was to induce tau accumulation in distal dendritic tips (Figures 3(c) and 5) [8, 9, 18]. This was accompanied by the loss of tubulin label that was most evident with tyrosinated tubulin in distal dendrites (Figures 5(e) and 6). By contrast, the level of tau expression had little effect on axonal tubulin levels (Figure 5(e)). The distal dendritic tau accumulations seen with high tau expression were always associated with dendritic degeneration (Figure 3(c)), which was confirmed when tau:tubulin ratios were calculated for GFP/DM1A immunolabeled sections using the ratio channel facility in Volocity (Figure 6(a)) and when ratio measurements were taken manually (Figure 6(b)). We found that the majority of sites at the POD (59%) showed tau:tubulin ratios of 8 : 1 or higher, with 24% at 12 : 1 or higher, whereas 82% of sites sampled 20–50 microns away from the POD showed ratios of under 7 : 1. Moreover, 90% of the sites with the highest tubulin intensity readings (i.e., with readings above neuropil immunostaining levels in the distal dendrites) were beyond 25 microns from the POD and had tau:tubulin ratios of 7 : 1 or below, while all of the sites with the highest tau readings were located at or distal to the POD (Figure 6). This was in close agreement with the Volocity ratio results and strongly indicates that some mechanistic connection exists between tau accumulation in the distal dendrites and localized MT loss and degeneration there.

### 3.7. Distally Transported Tau Is Disproportionately Phosphorylated at the 9G3 and 12E8 Sites

**Figure 7:**
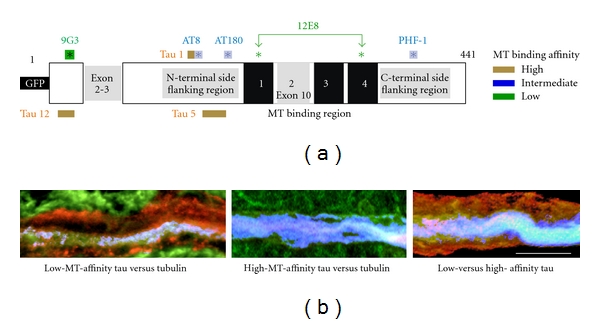
Low-versus high- MT-affinity tau species in ABC dendrites. (a) Schematic of the longest tau isoform showing tau phosphorylation sites. Phosphorylation of proline-directed sites flanking the MTBR (AT180, AT8, PHF1) produces a stoichiometrically graded reduction in MT affinity, whereas phosphorylation of KXGS sequences (S262, S356) within the MTBR region cause pronounced loss of MT binding affinity (12E8, green arrows). Phosphorylation of Y18 identifies fyn-mediated phosphorylation tau (9G3, shaded asterisk). Tau recognized selectively by mAbs specific to nonphosphorylated (Tau12, Tau5) and dephosphorylated (Tau1) sites is largely bound to MTs. (b) Illustration of the different spatial relationship between “low affinity” (9G3+ tau, left) and “high affinity” (Tau5+ tau, center). Tau is red channel, tubulin is green channel, and colocalized label (ImageJ colocalization highlighter threshold 50/50) is blue channel. Note that the central bundle of MTs in the left-hand image is relatively free of 9G3+ tau, whereas tubulin and Tau5 labels are extensively colocalized (center image—no red label is visible). Right image: Section is labeled for low-affinity (9G3, red channel) and high-affinity (Tau 1, green channel) tau. Note that there is considerable colocalization near the central MT bundle (blue channel), which is consistent with the dynamic nature of dendritic MTs. Scale bar: 10 *μ*m.

**Figure 8:**
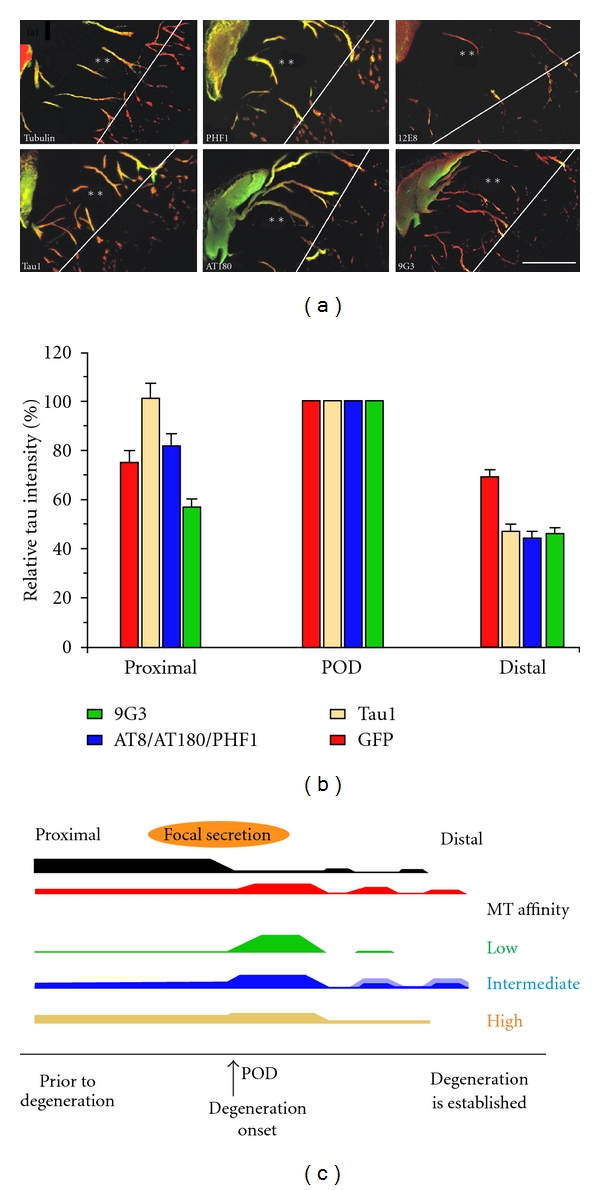
Evidence for the transport and distribution of hyperphosphorylated tau with low MT binding affinity into distal dendrites. (a) Examples of somatodendritic profiles from ABCs expressing high tau levels immunolabeled with phosphospecific Abs to epitopes on human tau (described in Figure 7) that identify low, medium, and high degrees of tau association with MTs. The green channel shows immunolabel Abs identifying tau with low (9G3, 12E8), medium (AT180, PHF1), and high (Tau1, Tau5, Tau12) MT affinity showing characteristically different distributions relative to the POD and to “total” tau distribution as shown by anti-GFP tag immunolabel (red channel of all images). Markers of non-MT-associated tau (9G3 and 12E8, right panels) are the most concentrated at the POD (line) and the least associated with MT-rich proximal dendrites (asterisks). By contrast, markers of dephosphorylated tau (e.g., Tau1—bottom left panel) are less concentrated at the POD and are largely codistributed with tubulin (top left), AT180 and PHF1, which mark intermediate states of MT affinity and show an intermediate distribution pattern. (b) Quantitation of the relationships illustrated by the sections shown in (a). Tau immunolabel intensity was normalized to the level seen at the POD for each antibody in each section examined and the relative intensity of immunolabel shown. Differences in distribution between proximal dendrites the POD and degenerating dendrites distal to the POD were highly significant for each antibody examined (all *P* values <0.005, Student's *t*-test). Markers of low-affinity tau (green) were significantly lower than medium-(blue) and high- (tan) MT-affinity markers in proximal dendrites (*P* < 0.01 and 0.001, resp.), whereas all tau markers declined to a level significantly below that of the POD in degenerating distal dendrites (all *P* values <0.001). Bars show standard error. (c) A schematic diagram summarizing the relationship between the distribution of tau with low (9G3, 12E8), medium (AT180, PHF1), and high (Tau1, Tau5, Tau12) MT affinity illustrated in (a) and analyzed in (b). Scale bars: (a) 100 *μ*m.

We found that tau phosphorylation state in distal dendritic accumulations is inversely correlated with respect to tubulin immunostaining levels and thus presumably not MT-associated (Figure 8). The tau in such accumulations is enriched in phosphotau species that are either incapable of binding (12E8+ tau [53–55]) or are not currently bound to MTs (9G3—see Figure 7). By contrast, markers identifying poorly phosphorylated tau with high MT affinity (Tau1, Tau5, Tau12) was localized to MT-rich regions (i.e., large dendrites), with mAbs marking proline-directed phosphorylation that reduces but do not abolish tau:MT binding (AT180, PHF-1 and AT8) showing an intermediate pattern (Figure 8). High-MT-affinity tau was largely colocalized with MTs in ABC dendrites and accumulated minimally at the POD, while 9G3+ tau or tau phosphorylated at KXGS sites in the MTBRs (12E8+ tau) was localized to either the POD itself or to a ventrolateral somatic location near the bases of large dendrites. Proline-directed phosphoepitopes (i.e., AT8, AT180, PHF1) were also more concentrated at the POD than was total (GFP+) tau (Figure 5(c)), but this tendency was less pronounced than with 9G3 and 12E8 (Figure 8).

### 3.8. Tau Phosphorylated at the 9G3 and 12E8 Sites Is Associated with Fyn- and LC3-Containing Vesicles near Secretion Sites in ABC Dendrites

**Figure 9:**
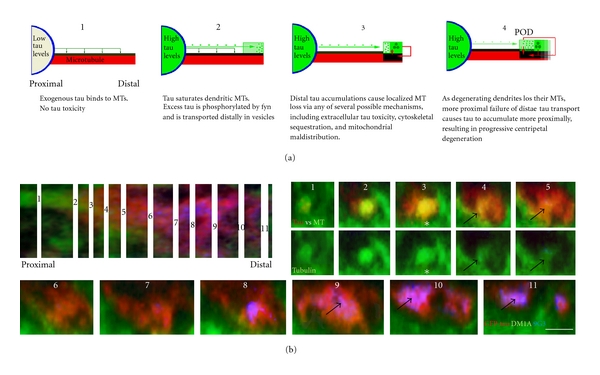
The mechanism of tau-induced dendritic degeneration in ABCs. (a) Schematic summarizing the hypothesized sequence of events leading to tau-induced degeneration in ABC dendrites. Low-level exogenous tau expression (1) does not saturate the tau:tubulin binding sites on dendritic MTs, permitting all exogenous tau to bind directly to dendritic MTs (green arrows) and remain poorly phosphorylated and nontoxic. (2) In ABCs expressing enough exogenous tau to saturate existing MTs, excess tau becomes phosphorylated at the 9G3 and 12E8 sites and associated with trafficking vesicles. These are transported distally to and accumulate in the relatively MT-poor dendritic tips, where they induce degeneration, possibly by being secreted to form toxic extracellular deposits. (3) The consequent increased intracellular Ca^2+^, MT destabilization, misdistribution of mitochondria, and cytoskeletal aggregation become self-perpetuating (4, red arrows), driving progressive dendritic degeneration (black). (b) Colocalization of MT loss and non-MT-associated tau accumulation. A *z*-axis reconstruction of a dendrite (left) containing a bundle of MTs along which tau was being transported distally (1–5) and accumulating (6–11) at the POD. The distribution of total tau (GFP tag, red) and non-MT-associated tau (9G3, blue) is shown versus tubulin (green). Numbers correspond to the sites in the original stack (left) chosen for cross-sectional views (right and bottom). Note that MTs (asterisks, 3) rapidly become more saturated with tau between sections 1 and 3. Above the tau-MT saturation point (3, caret), the MT bundles lose tubulin label over a very short distance (black arrows, 4 and 5). Scale bar: 2 *μ*m.

**Figure 10:**
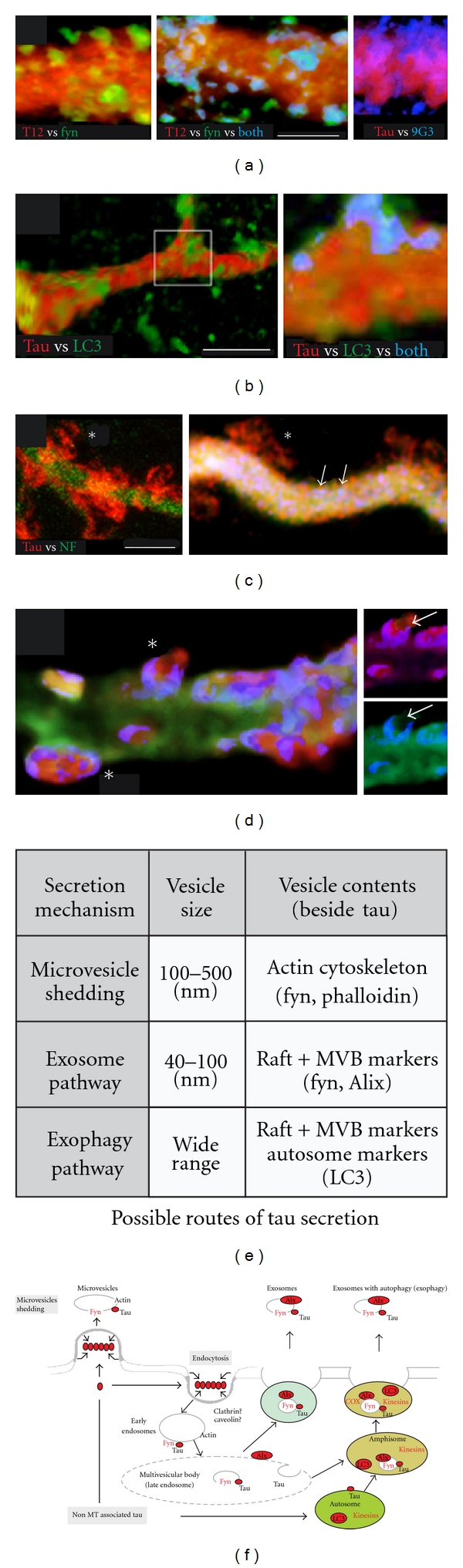
High-resolution confocal images of “focal” tau secretion from ABCs. (a) High magnification imaging of surface rendered (ImageJ 3D Viewer) images of dendrites near the POD show significant colocalization between fyn and tau (left) in what appear to be exocytotic profiles (yellow). Active tau secretion via exocytosis can be identified in high-magnification confocal images of the surfaces of dendrites proximal to the POD in conjunction with the secretion of fyn-positive vesicles. Selective inclusion of 9G3 in secreted tau can be seen in exocytosing profiles from intact dendrites near the POD (right). (b) The autophagy marker LC3 undergoes secretion from ABC dendrites in a process that appears largely independent of tau (left), but higher resolution images show low levels of tau found in LC3+ vesicles that appear to be undergoing exocytosis (blue). (c) The gross appearance of tau leaving ABC dendrites suggests secretion via a membrane “shedding” mechanism (asterisks). Note that, in the right-hand image, this is not associated with the release of 9G3+ tau (vesicles, arrows), but rather with GFP-tau (red), which selectively colocalizes with exocytotic profiles (left, center, asterisks) (d) The main panel of d shows a dendrite triple-labeled with phalloidin (f-actin) and both total (anti-GFP-tag—red) and Tau12+ tau (blue). Only the phalloidin label that colocalized with tau is shown (colocalization highlighter, ImageJ), revealing phalloidin-tau+ vesicles that appear to be undergoing exocytosis (asterisks). When Tau12 and GFP channels are separated (insets), the relative exclusion of MT-associated tau relative to the GFP-tau from exocytosis profiles is evident (arrows). (e) While tau lacks typical features (signal peptide, acylation sites) of proteins that undergo conventional secretion, the well-established association of tau with fyn and other lipid raft-associated tyrosine kinases suggests some “unconventional” secretion mechanisms for tau protein, including both microvesicular and exosomal routes, both of which could involve cosecretion of tau and fyn. (f) The images shown in (a–d) suggest that multiple unconventional secretion pathways are involved in “focal” tau secretion from ABC dendrites. These include the direct shedding of microvesicles containing raft proteins (left), the generation of multivesicular bodies via raft “patching” and endocytosis, followed by MVB exocytosis and exosome release (center), and a recently identified variant of the exosomal pathway (exophagy) which involves autophagosomal fusion before exosome release (right). Scale bars (a) 5 *μ*m, (b) 20 *μ*m, (c) 5 *μ*m.

In earlier studies, we showed that distal dendrites of high-expressing ABCs become strongly tau positive [9] and filled with vesicular inclusions [8, 18]. Some of the tau accumulated at dendritic tips or the POD becomes transiently immunopositive for 9G3 (marking tau phosphorylation at Y18 by srk-family tyrosine kinases) and may undergo secretion to the extracellular space [43]. In this study, we characterized these inclusions in more detail by immunolabeling sections through tau-secreting ABCs with multiple anti-tau antibodies (Figures 7 and 8) together with Abs to specific vesicular markers, as well as the fluorescently labeled f-actin marker phalloidin. We found that tau-positive vesicular profiles at the surfaces of intact dendrites near the POD in ABCs expressing high levels of tau were colocalized with either non-MT-associated tau epitopes (9G3, 12E8) and/or markers of vesicular organelles (Figures 9 and 10). At sites where secretion was ongoing, many but not all 9G3 tau+ vesicles were colocalized with the srk family tyrosine kinase fyn, which can be considered a dendritic endosome marker [56, 57], with 12E8-positive tau being similarly distributed (not shown). When compared to markers of high-MT-affinity tau (Tau5, Tau12), the GFP tag fused to the tau amino terminus also selectively identified exocytotic profiles at sites of focal secretion (Figure 10).

## 4. Discussion

The misprocessing of tau is a key event in neurodegenerative tauopathies and is typically not associated with MTs. However, the details of the toxicity-associated tau misprocessing pathway in the absence of MT binding are still very poorly understood. We employed overexpression as a means of generating non-MT-associated tau in ABCs in order to characterize this pathway and its relationship to tau-induced degeneration in a high-resolution cellular model *in situ*. We show that the expression of exogenous tau causes a dose-dependent loss of somatodendritic MTs that is spatiotemporally correlated with tau-induced degeneration. We confirm earlier findings in the lamprey system and elsewhere that tau colocalizes with certain vesicular organelles, especially when phosphorylated at Y18 [18, 36]. Our results indicate that the saturation of dendritic MTs by exogenous human tau accounts for the changes in tau distribution and phosphorylation seen between ABCs expressing low and high levels of tau. Tau expression above this threshold is correlated with degeneration patterns that account for all of the characteristics of tau-induced toxicity observed in this model (and others to our knowledge) so far. We also conducted the first detailed morphological characterization of tau secretion from CNS neurons in an *in situ* tauopathy model and present evidence that tau secretion occurs via an unconventional mechanism with characteristics of microvesicle and exosome-mediated routes. These events are summarized in Figures 9 and 10, and their relevance to and implications for tau pathobiology in human disease are discussed below.

### 4.1. Distribution of Exogenous Tau in ABCs

The overexpression of both WT and tauopathy mutant tau in ABCs in most cases produces phosphorylated tau accumulation in dendrites, as reported previously [8, 9, 18, 41]. While there did appear to be some preferential distribution of exogenous tau to the axon at relatively low levels of overexpression in ABCs, this effect was slight and was apparently not related to the preferential association of tau with axonal versus dendritic MTs. Moreover, even this slight preference was lost with relatively moderate levels of overexpression, a pattern similar to that seen with early murine transgenic models [58, 59]. This is consistent with reports that active targeting of tau to the axon occurs mainly at the mRNA rather than the protein level [60, 61] and with reports that tau may be slightly overexpressed in non-AD human tauopathies [62]. We, therefore, propose that tau overexpression in ABCs re-creates the circumstances needed for tau toxicity (e.g., tau hyperphosphorylation, tau cleavage) simply by exceeding the tau-binding capacity of endogenous dendritic MTs. The relative insensitivity of axonal MTs to the destabilizing effect of tau overexpression suggests that the primary effect is a gain of a toxic function in the dendrites rather than the loss of normal MT stabilization axon in the axon.

### 4.2. MT Saturation and the Centrifugal Redistribution of Non-MT-Associated Tau

In the ABC model, the occurrence of tau-induced dendritic MT loss, vesicular tau, and tau secretion at the POD appear to be mechanistically related given that the POD is also the site of both the highest focal tau secretion levels and of the sharpest decrease in dendritic tubulin levels (Figure 5(b)). Our findings are also consistent with studies showing tau redistribution to MT-poor regions of the cell whenever it is present in excess of available MT tau binding capacity, whether this occurs by overexpression or by some disease-related mechanism that limits tau:MT binding [63]. The limited tau binding capacity of dendritic MTs for tau in ABCs thus appears to serve as a threshold above which excess tau is processed by an alternative, toxicity-generating pathway. Moreover, since tau has been shown to modulate the activity of MT-associated motor proteins that mediate dendritic transport [64, 65], often in a disease-specific [66] or overexpression-induced [67] manner, it seems likely that toxicity resulting from tau accumulation at localized dendritic loci may have relevance to the pathogenesis of human tauopathies, which typically feature abnormal somatodendritic phosphotau accumulation.

### 4.3. A Significant Proportion of Non-MT-Bound Tau Is Vesicle Associated and Is Associated with Tau Toxicity

Hyperphosphorylated, non-MT-associated tau is widely considered to be the agent of neurodegeneration in human tauopathies, although little is known about its underlying toxicity mechanism. Here we confirm other demonstrations in the ABC model that a tight correlation exists between 9G3 phosphorylation and localized tau-induced neurodegeneration [41] (Figures 7 and 9), and that tau overexpression is associated with vesicle accumulation in ABC distal dendrites [8, 18]. Our observations show that the phosphorylated tau accumulating where dendritic MTs are being lost in ABC dendrites is largely vesicle associated and that some of these vesicles resemble endosomes and/or autophagosomes in their general appearance and colocalization with endosome (fyn) and autophagosome (LC3) markers (Figure 10). Moreover, this toxic, vesicle-associated tau accumulates selectively in MT-poor regions that are adjacent to more proximal dendritic segments containing organized MT bundles (Figure 9), suggesting (1) that it is translocated via MT-mediated transport in ABC dendrites and (2) that its accumulation is both the cause and the consequence of localized MT destabilization. We propose that abnormal tau involvement in the formation of trafficking vesicles originating from the plasma membrane and/or the autophagy pathway in the absence of MT association cause the accumulation of 9G3/12E8, positive tau, which in turn destabilizes nearby MTs via an as yet unknown mechanism. This could account for the selective MT loss associated with neurofibrillary pathology in humans [1, 2, 68, 69] and suggests that hyperphosphorylated, non-MT-associated tau may be bound to vesicles that are transported within the cell via MT-mediated mechanisms in human tauopathies. This idea has been little explored but is consistent with much of the relevant human tauopathy literature and could add a new dimension to our conception of abnormal tau processing in neurodegenerative disease. Neuropathological studies suggest that hyperphosphorylated tau may oligomerize into granular and ultimately fibrillar deposits and that an unknown element in this sequence of events mediates tau toxicity [3, 68, 69]. This is supported by demonstrations that “pretangle” deposits of granular, hyperphosphorylated tau precede NFT formation in murine models [70, 71] and appear to do so in humans [68, 69]. However, interactions between tau and membrane/vesicle-associated proteins are also associated with and may mediate aspects of neurofibrillary disease. Tau oligomerization can be driven by fatty acids *in vitro *[72, 73], while NFT formation is associated with membranous organelles *in vivo *[74, 75]. Misprocessed tau has been associated with abnormalities in several types of vesicular organelle in AD and tauopathy models, including the Golgi apparatus [39], lysosomes [76–78], autophagosomes [76, 79–81], and most recently exosomes [82]. The last two are of particular interest in light of our findings here that vesicular tau is colocalized with the lipid raft-associated tyrosine kinase fyn, which itself is both required for extracellular beta-amyloid-mediated toxicity via tau [83, 84] and is mislocalized in AD [85, 86]. Since fyn has been identified in exosomes (along with other signal transduction associated kinases) [87], it appears that interaction with and phosphorylation of tau at the 9G3 site by fyn may be a key feature of both secretion and toxicity-associated tau processing. Fyn activation during signal transduction typically causes the oligomerization and endocytosis of downstream elements [88] and targets at least some oligomerized targets of fyn phosphorylation to exosomes [89, 90], a process which appears to be highly analogous to what we see with tau in the ABC system. Similarly, the involvement of the (macro)autophagy pathway of protein turnover in AD has long been known [79–81, 91], and this has more recently been extended to non-AD tauopathies [77–79], where it now appears to play a largely disruptive role in autophagic protein turnover mechanisms that may well exacerbate tau toxicity in these diseases [80]. The association of non-MT-associated tau with fyn or LC3-positive is consistent with microvesicular or exosomal secretion, as discussed below.

### 4.4. Possible Mechanisms of Dendritic Tau Secretion

In a previous study, we found that tau secretion from ABC dendrites requires elements in both the tau N-terminal and MTBR domains and inferred that such secretion is transient, first appearing before the onset of overt degeneration and ending before complete fragmentation of the dendrite occurs [41]. While these results showed tau secretion to be an active biological process rather than a passive release of tau from autolyzing neurons, they gave little indication as to the mechanism of tau secretion and why it is spatiotemporally correlated with dendritic degeneration in ABCs. The present study yields specific indications as to the secretion mechanism by (a) correlating MT loss and the distribution of non-MT-associated tau with “focal” secretion from ABC dendrites just before the onset of dendritic degeneration and by characterizing the (b) morphology of exocytotic profiles and (c) the phosphorylation state and proteins colocalized with the secreted tau. In particular, we show that at least some tau secretion is associated with fyn-positive, endosome-like vesicles in which tau has been phosphorylated at the site (Y18) characteristically phosphorylated by fyn, and that another subset of tau-containing vesicles is colocalized with the autophagy marker LC3, which also appears to be exocytosed. It is noteworthy that, in normal autophagy, LC3 is rarely seen among the exocytosed proteins due to its exposure to lysosomal hydrolases [80]. However, tau appears to interfere with autophagy generally in tauopathies and with the acidification of autolysosomes in particular. A third subset of externalized tau appears to be morphologically dissimilar from vesicle exocytosis, suggesting a mechanism akin to plasma membrane blebbing [92] or the “membrane shedding” seen with the unconventional secretion of galectin-1 [90] (Figure 10). A more general issue relevant to both dendritic tau secretion and toxicity was raised by a recent study by Ittner et al. [93] which showed that dendritic localization of tau can selectively mediate Abeta toxicity. This is consistent with the generally dendrite-specific toxicity observed in this study and suggests that the abnormal juxtaposition of tau and dendrite-specific signal transduction mechanisms might play a role in both tau-mediated toxicity and tau secretion.

## 5. Conclusion

Little is currently known about exactly how non-MT-associated tau becomes cytotoxic en route to its oligomerization and polymerization into neurofibrillary deposits in human tauopathies other than that (a) tau becomes a substrate for tyrosine as well as serine/threonine kinases and (b) tau undergoes progressive amino and carboxyl terminal cleavage at some point in the process. Here we show that, when tau becomes disassociated from MTs by virtue of overexpression, it is processed via a novel pathway that generates localized cytotoxicity manifested by MT loss and dendritic degeneration that is preceded by the secretion of some of the misprocessed tau via an unconventional mechanism that involves elements of both exosome and autophagy pathways. Tau misprocessing in ABCs involves (1) the interaction of tau with elements in the subcortical cytoskeleton and its phosphorylation of tau at characteristic AD-related sites, (2) the MT-mediated transport of hyperphosphorylated tau in association with vesicles and their accumulation in distal dendrites, and (3) the progressive MT loss and dendritic dysfunction in the immediate vicinity of these deposits, which may be exacerbated by localized secretion followed by extracellularly mediated toxicity. While the roles played by these events in the generation of neurofibrillary deposits, toxic tau oligomers, and the interneuronal spread of tau lesions are still unclear, our results lay the ground for acquiring a more detailed understanding of the role played by tau misprocessing in the cytopathogenesis of neurodegenerative disease.
